# Identification of an intrusive-hypervigilant phenotype of posttraumatic stress symptoms with unique stress peptide and amygdala functional connectivity profiles

**DOI:** 10.1038/s41386-026-02396-0

**Published:** 2026-04-08

**Authors:** Kevin J. Clancy, Caitlin Ravichandran, Sydney A. Jobson, Victor May, Sayamwong E. Hammack, William A. Carlezon, Kerry J. Ressler, Scott L. Rauch, Isabelle M. Rosso

**Affiliations:** 1 Division of Depression and Anxiety Disorders, McLean Hospital, Belmont, MA, USA; 2 Department of Psychiatry, Harvard Medical School, Boston, MA, USA; 3 Larner College of Medicine, University of Vermont, Burlington, VT, USA; 4 Department of Psychology, University of Vermont, Burlington, VT, USA

## Abstract

Posttraumatic stress disorder (PTSD) is a highly heterogeneous psychiatric disorder, complicating efforts to identify consistent biological markers and develop targeted treatments for individuals exposed to trauma. Recent research has identified a distinct intrusive-hypervigilant (IH) phenotype, which is characterized by heightened intrusive reexperiencing and hypervigilance symptoms along with elevated levels of pituitary adenylate cyclase-activating polypeptide (PACAP), a neuropeptide involved in stress response via amygdala signaling. In an independent sample of 172 symptomatic trauma-exposed adults, we replicated this IH phenotype using latent profile analysis of Clinician-Administered PTSD Scale for DSM-5 symptom severity ratings and expanded its biological characterization using resting-state functional magnetic resonance imaging (rs-fMRI). Consistent with prior work, the identified IH group demonstrated more severe intrusive reexperiencing (Cohen’s d’s = 0.61–6.93) and hypervigilance symptoms (d’s = 0.57–0.88) and higher PACAP levels compared to groups with generally High (d = 0.35) or Low (d = 0.44) symptom severity. Additionally, the IH phenotype exhibited stronger functional connectivity of the centromedial, but not basolateral, amygdala with regions in the occipital cortex (d’s = 0.78–0.95), precuneus (d’s = 1.20–1.21), and medial prefrontal cortex (d’s = 0.81–1.18)—areas primarily within the Default Mode and Visual Networks. Meta-analytic decoding linked these regions to mental imagery, memory processing, fear, and threat perception. These findings support the existence of an IH phenotype of posttraumatic stress that may exhibit a distinct biological profile, characterized by exaggerated interactions between memory, threat, and arousal systems that may be mediated by PACAP and its effects on amygdala connectivity. This phenotype may serve as a promising target for precision psychiatry approaches, including pharmacological and neurotherapeutic interventions that modulate PACAP signaling and amygdala connectivity.

## INTRODUCTION

Posttraumatic stress disorder (PTSD) is a markedly heterogeneous psychiatric disorder, with potentially 636,129 different combinations of 20 symptoms across four distinct symptom clusters that fulfill DSM-5 diagnostic criteria for PTSD [1, 2]. Beyond the diagnosis of PTSD, a significant number of trauma-exposed individuals report clinically significant distress and impairment through the endorsement of PTSD symptoms that do not meet full DSM-5 diagnostic criteria (i.e., subthreshold PTSD) [3]. Combined, this vast clinical heterogeneity hinders efforts to identify reliable biological substrates that are needed to advance mechanism-based therapeutics of posttraumatic stress.

The heterogeneity of posttraumatic stress has motivated the identification of “subtypes”, or distinct clinical phenotypes that may have more homogeneous biological mechanisms that can be reliably targeted under a precision psychiatry framework. However, to date, the emergence of reliable subtypes remains elusive. A dissociative subtype of PTSD is the only recognized diagnostic subtype based on DSM-5 criteria and is characterized by the additional endorsement of dissociative symptoms beyond the “classic” 20 PTSD symptoms. Additionally, the ICD-11 recognizes Complex PTSD as a distinct diagnosis that adds disturbances in self-organization to core PTSD symptoms [4]. Beyond these recognized subtypes, additional work has offered evidence for other subtypes based on a predominance of internalizing versus externalizing or fear versus dysphoria symptoms [5–9]. However, other work suggests that the most common subgroups across latent profile and class analyses of PTSD symptoms are “low”, “medium”, and “high” symptoms [10, 11].

Importantly, much of this work has utilized samples of participants with shared or similar traumatic experiences. This approach may fail to capture the diverse etiology of PTSD, as different types of traumatic experiences (e.g., single versus recurrent, interpersonal versus non-interpersonal) may result in different symptoms and neurobiological alterations [12–15]. Moreover, prior latent class and profile analyses focus on samples meeting full diagnostic criteria for PTSD. This overlooks the significant number of trauma-exposed individuals who experience clinically-significant distress and impairment but do not meet diagnostic thresholds for all four symptom clusters [3].

Addressing these concerns in a diverse, trauma-exposed sample with subthreshold and threshold PTSD, Adams et al. [16] recently identified a distinct clinical phenotype of posttraumatic stress that was marked by specific elevations in intrusive reexperiencing and hypervigilance symptoms [16]. Moreover, they demonstrated that the intrusive-hypervigilant (IH) clinical profile had impaired fear extinction retention. This clinical phenotype aligns with prior work demonstrating a link between arousal processes and intrusive trauma memories: hyperarousal symptoms predict later reexperiencing symptoms [17–19], experimental manipulation of physiological arousal triggers the reactivation of aversive memories [20, 21], and aversive memories are coupled with elevations in sympathetic arousal [22–26]. At the neurobiological level, arousal facilitates reinstatement of salient memory information via increased activity of locus coeruleus (LC) and amygdala pathways that enhance the expression of prioritized memory representations in cortical regions [27]. Conceptual models further propose that LC and amygdala circuit activation leads to rapid reactivation in sensory-perceptual cortices [28–30], such that retrieval under high arousal leads to rapid and involuntary reactivation of sensory memory traces [31].

Consistent with the neurobiological link between arousal and memory processes, Adams et al. [16] further demonstrated that the IH clinical phenotype had robust elevations in circulating levels of pituitary adenylate cyclase-activating polypeptide (PACAP). PACAP is a highly-conserved neuropeptide that serves as a critical regulator of stress response, including the activation of physiological arousal and defensive responding through signaling within the amygdala complex, particularly the central amygdala [32–39]. It has been linked to the pathophysiology of PTSD through alterations in amygdala activity and subsequent effects on arousal, threat processing, and fear memory [40, 41]. Recent evidence further links PACAP to exaggerated functional connectivity (FC) of the amygdala with regions of the default mode network (DMN), a network frequently implicated in PTSD through its role in autobiographical memory, arousal, and internally (versus externally) oriented attention [42]. Additional evidence suggests that PACAP’s effects are stronger in female than male patients, indicating that it may reveal mechanistic insights into sex differences in PTSD risk and prevalence [16, 40]. Correspondingly, Adams et al. demonstrated that the IH subtype with elevated PACAP was predominantly female, which is consistent with prior work highlighting interactions between estrogen and PACAP systems in conferring risk for PTSD [40]. This suggests a biological phenotype of posttraumatic stress that is tied to biological sex.

Overall, findings from Adams et al. reveal a potentially more homogenous clinical-biological subgroup that pairs elevated intrusive reexperiencing and hypervigilance symptoms with biological and psychophysiological markers of fear memory and arousal. This may represent an important step towards a biologically-grounded clinical subtype to target through mechanism-based interventions in a precision psychiatry approach.

The current study sought to replicate findings of an IH clinical phenotype with elevations in PACAP and build upon their biological profile by providing novel evidence for distinct patterns of amygdala FC. Using resting-state functional magnetic resonance imaging (rs-fMRI) in an independent sample of symptomatic trauma-exposed adults, we tested the hypothesis that an IH phenotype would be associated with exaggerated FC of the amygdala with networks implicated in trauma memory and arousal. Consistent with prior work, we hypothesized these effects would be specific to the right central amygdala, where PACAP-resting state associations are strongest [42].

## METHODS

### Participants

One-hundred and seventy-two trauma-exposed adults were recruited and enrolled via advertisements in the local community. This sample is inclusive of the sample reported in previously published work examining the association between PACAP and amygdala FC in trauma-exposed adults, along with additional participants recruited after the initial publication [42]. Study procedures were approved by the Mass General Brigham Human Research Committee, and all participants provided written informed consent. Participants completed a fasting blood draw, clinical interview, self-report questionnaires, and a 13-min eyes-open resting-state fMRI scan. Participants were included if they met DSM-5 diagnostic criteria for at least two PTSD symptom clusters based on the Clinician Administered PTSD Scale for DSM-5 (CAPS-5), constituting subthreshold (n = 57; 33%) and threshold (n = 115; 67%) PTSD [3]. Given a prior interest in biological sex differences motivated by prior work [43], participants were required to be the same sex as assigned at birth, female participants were to be premenopausal, and participants with a history of receiving hormonal replacement therapy or undergoing surgery to change biological sex were excluded. Other exclusion criteria are detailed in Clancy et al. [42].

Of the 172 enrolled participants with complete clinical interview data, 158 (106 female) had detectable serum PACAP levels (excluded: undetectable values = 13, levels below reliable detection threshold = 1). A total of 134 (85 female) had usable fMRI data (excluded: excessive motion = 14, incomplete scanning = 7, poor structural-functional alignment = 4, significant artifact = 6, no scanning performed = 7). Demographic and clinical characteristics of the sample are provided in Table 1.

### Clinician-administered PTSD scale for DSM-5 (CAPS-5)

The CAPS-5 [44], the gold-standard diagnostic interview for PTSD, was administered by doctoral-level clinicians. This interview consists of 30 items designed to assess the onset, duration, and impact of PTSD symptoms, yielding a determination of PTSD diagnosis and symptom severity. Specifically, each of the 20 DSM-5 PTSD symptoms is given an overall severity rating, which reflects the intensity and frequency of the symptom within the past month. Severity ratings for each symptom are made using a 0–4 Likert Scale (0 = Absent, 4 = Extreme/incapacitating).

### PACAP

Details about PACAP collection and analysis are provided in the Supplementary Methods and Clancy et al. [42]. Fourteen samples were excluded due to undetectable (n = 13) or unreliably low (n = 1) PACAP levels, resulting in a final sample of n = 158 for PACAP analyses. Consistent with Adams et al. [16], PACAP values were square-root-transformed to reduce data compression and correct a left-skewed distribution.

PACAP levels were analyzed in two batches. There was a significant effect of Batch on PACAP levels (Batch 1 mean = 29.53, Batch 2 mean = 20.98; t = 3.08, p = 0.003), with unequal variances across the two batches (Levene’s Test F = 6.22, p = 0.014). These Batch differences were not explained by clinical or demographic variables; therefore, Batch (binary 1 or 2) was included as a covariate in all PACAP analyses.

### Latent symptom profile analysis

Gaussian mixture modeling was performed using the Mclust package in R [45] to identify latent symptom profiles using symptom severity ratings from the 20-item CAPS-5 interview. Specifically, we aimed to replicate the IH symptom profile identified in Adams et al. [16] through a complementary yet distinct approach to demonstrate the external reliability of this symptom profile, choosing to utilize latent profile over latent class analysis to retain information gained from the continuous nature of CAPS-5 symptom severity data. Four models with different covariance structures examined a range of 1 to 4 profiles. Optimal model fit was determined using the Bayesian Information Criterion (BIC). Additional details are reported in the Supplementary Methods.

### MRI data acquisition and preprocessing

Imaging was conducted at the McLean Hospital Imaging Center on a 3T Siemens Prisma scanner. MRI data were preprocessed using fMRIPrep version 20.2.7 [46]. Fourteen participants were excluded for excessive motion, 7 had incomplete scanning, 4 had inadequate alignment of structural and functional scans, 6 had significant imaging artifacts, and 7 had no scanning performed, resulting in a final sample of n = 136 for fMRI analyses. Notably, participants excluded from fMRI analyses did not differ from those included on any demographic, clinical, or biological variables of interest (Supplementary Table 1). Protocol details and additional denoising steps are reported in the supplement and Clancy et al. [42]. Cleaned timeseries from these preprocessing and denoising steps were subsequently submitted to resting-state functional connectivity analyses.

### Resting-state functional connectivity analyses

Seed-based resting state connectivity analyses were conducted in CONN using cleaned timeseries of BOLD signal extracted from left and right (l/r) centromedial (CMA) and basolateral (BLA) amygdala regions of interest (ROIs) generated from the JuBrain Atlas (Supplementary Fig. S1) [47, 48]. Whole-brain seed-based correlations were performed for l/rCMA and l/ rBLA seeds to compute whole-brain FC maps. Seed-based FC values were Fisher’s z-transformed prior to further statistical analyses.

### Analytic plan

Given our specific a priori interest in an IH symptom profile, an identified group matching this profile was compared to other symptom groups to determine the clinical and biological correlates of this IH phenotype. First, the identified IH group was compared to all other symptom profiles combined into a single non-IH group, as prior findings showed no PACAP differences between non-IH clinical phenotypes [16]. This approach was contingent on the absence of any statistically significant difference between the non-IH clinical phenotypes across biological markers of interest in our sample. Comparisons of the IH group versus the single non-IH group were followed by separate comparisons between the IH group and other groups individually.

Categorical characteristics were compared between IH and non-IH phenotypes using Chi-squared tests of proportions for sex assigned at birth (male vs. female) and PTSD diagnosis (yes vs. no) using the proportions test function in R [49], testing for higher female representation and PTSD diagnosis rates in the IH group. Model-based estimated marginal means in PACAP levels by phenotypic group, and test statistics and p-values for associated significance tests, were calculated from results of linear regression models controlling for Batch using the emmeans package for R [50]. Robust standard errors were used for all analyses examining PACAP with Batch as a covariate due to unequal variances between batches. Complementary mixed-effects models with Batch as a random effect are presented in the Supplementary Results; primary analyses treated batch as a fixed effect to avoid unstable model fit associated with very few levels of random effects (ex., 2 batches) [51]. T-test contrasts examined Group differences in whole-brain seed-based amygdala FC maps. Whole-brain FC results were thresholded at p < 0.005 (uncorrected height threshold), p < 0.0125 FDR-corrected cluster threshold, to correct for multiple comparisons across the 4 amygdala ROIs (0.05/4 ROIs = 0.0125). The SPM Anatomy Toolbox was used to assign estimated anatomical labels for significant clusters [52]. Meta-analytic decoding of identified clusters was performed using the NiMARE v0.0.11 package, which ascribes functional labels to images using the NeuroSynth meta-analytic database [53–56].

Finally, we tested associations between clinical symptoms and biological markers. Individual linear regressions controlling for Batch examined associations between PACAP levels and symptoms that differentiated the IH group from the non-IH group. Pearson correlations examined associations between symptom severity and amygdala FC. Symptom associations were corrected for multiple comparisons using family-wise error (FWE) correction across all examined symptoms.

One-tailed tests were used for replication analyses (e.g., elevated PACAP in IH), and two-tailed tests for novel analyses (e.g., FC differences between groups, symptom associations with identified biological markers). One-tailed p-values are explicitly labeled in the results and are accompanied by two-tailed p-values; all other p-values are two-tailed. Given a priori findings that implicate biological sex as an important factor of the IH phenotype and its underlying neurobiology [16, 40], sex assigned at birth was not included as a covariate in models; however, models incorporating sex assigned at birth are presented in the Supplementary Results.

## RESULTS

### Latent profile analysis

Consistent with Adams et al. [16], a three-profile model of CAPS-5 symptoms provided the best fit based on the BIC. The resulting profiles included: a High group (n = 73) characterized by elevated severity across most PTSD symptoms; a Low group (n = 61) with generally low symptom severity; and an Intrusive-Hypervigilant (IH) group (n = 38) marked by differential elevations in intrusion and hypervigilance symptoms. Compared to the High and Low groups, the IH Group demonstrated significantly greater severity of trauma-related intrusive memories (B1; IH > High/Low: t = 3.04/ 6.38, p = 0.008/ < 0.0001, Cohen’s d = 0.61/1.32), flashbacks (B3; IH > High/Low: t = 34.17/33.55, p’s < 0.0001, d = 6.83/6.93), and hypervigilance (E3; IH > High/Low: t = 2.87/4.27, p = 0.013/0.0001, d = 0.57/0.88; Fig. 1). Complete descriptive statistics for all CAPS-5 symptoms are reported in Supplementary Table 2.

Also in line with prior findings, the IH group had a greater proportion of females (79%) compared to the High (63%; IH > High χ2 = 2.94, p = 0.043) and Low groups (52%; IH > Low χ2 = 7.02, p = 0.004). The High and Low groups did not differ in sex composition (High ≠Low χ2 = 2.94, p = 0.217). We also observed differences in the proportion of threshold (vs. subthreshold) PTSD diagnoses, with the IH group having a greater proportion (79%) compared to the Low group (36%; IH > Low χ2 = 17.27, p < 0.001) but not the High group (86%; IH > High χ2 = 0.99, p = 0.841). The High group also had significantly more full PTSD cases than the Low group (High > Low χ2 = 36.16, p < 0.001).

### PACAP38

There were no significant differences in PACAP levels between the High (M = 24.02, SE = 1.63) and Low groups (M = 22.86, SE = 1.77; t(120) = 0.52, p = 0.607), justifying their combination into a single non-IH comparison group. The IH group (M = 29.08, SE = 2.53) had significantly higher PACAP levels compared to the combined non-IH group (B = 0.38, SE = 0.18, t(155) = 1.97, p = 0.025 one-tailed, p = 0.050 two-tailed, d = 0.39). Follow-up contrasts confirmed elevated PACAP levels in the IH group compared to both the High (t(154) = 1.65, p = 0.050 one-tailed, p = 0.101 two-tailed, d = 0.35) and Low groups (t(154) = 2.04, p = 0.025 one-tailed, p = 0.050 two-tailed, d = 0.44; Fig. 2).

### Functional connectivity

Whole-brain rCMA-seed connectivity maps confirmed no differences between the High and Low groups, again supporting their combination into a single, non-IH group. Relative to this combined non-IH group, the IH group exhibited stronger rCMA connectivity with three midline clusters (Fig. 3A): a cluster spanning the parietooccipital sulcus and intracalcarine cortex (k = 302 voxels, cluster FDR q = 0.001, peak MNI = [−4, −72, 16], T = 4.15), a precuneus cortex cluster (k = 287, cluster FDR q = 0.001, peak = [−4, −56, 68], T = 4.73), and a medial prefrontal cortex (mPFC) cluster comprising the paracingulate gyrus and anterior cingulate cortex (ACC; k = 210, cluster FDR q = 0.005, peak = [−2, 46, 14], T = 4.63). Mapping these clusters onto Yeo’s 7-network atlas (Fig. 3B) situated them predominantly within the Default Mode Network (k = 212) and Visual Network (k = 208), and to a lesser extent the Dorsal Attention (k = 99), Ventral Attention (k = 81), Somatomotor (k = 76), and Frontoparietal (k = 46) networks. Follow-up group contrasts revealed the IH group demonstrated greater rCMA FC across all three clusters compared to both the High and Low groups (Fig. 3C and Supplementary Table 3). No significant group differences emerged for seed connectivity from the rBLA, lCMA, or lBLA.

### Associations with clinical symptoms

Across all participants, PACAP levels were modestly associated with CAPS-5 item severity scores for flashbacks (β = 0.17, SE = 0.08, p = 0.025; Fig. 4A), which did not survive FWE correction for multiple comparisons across the three examined symptoms. PACAP levels were not associated with trauma-related intrusive memories (β = 0.08, SE = 0.08, p = 0.336) or hypervigilance (β = 0.02, SE = 0.08, p = 0.787). Flashback severity was additionally associated with rCMA-POS (r = .35, p < 0.0001; Fig. 4B), rCMA-Precun (r = 0.43, p < 0.001; Fig. 4C), and rCMA-mPFC FC (r = 0.37, p < 0.0001; Fig. 4D). Additional weak and trending associations were seen between rCMA-mPFC FC and hypervigilance (r = 0.17, p = 0.048), as well as intrusive memories (r = 0.15, p = 0.079; Fig. 4D), which did not survive FWE correction for multiple comparisons.

## DISCUSSION

In an independent sample of trauma-exposed adults with symptoms spanning subthreshold to threshold DSM-5 PTSD, we replicated the findings of Adams et al. [16], identifying an IH clinical phenotype characterized by more severe intrusive memories, flashbacks, and hypervigilance, as well as elevated levels of circulating PACAP. Extending prior work, we provide novel evidence that this IH phenotype is associated with stronger rCMA FC with midline cortical regions, particularly within the default mode and visual networks. Importantly, both PACAP levels and rCMA FC were significantly associated with more severe flashbacks, supporting their potential as biomarkers for this clinical phenotype.

Our identification of an IH clinical phenotype aligns with existing neurocognitive models of PTSD symptoms that highlight the intersection of arousal, memory, and attention. The “warning signal” hypothesis of trauma reexperiencing symptoms posits that neutral sensory cues can elicit exaggerated arousal and trigger intrusive memories or flashbacks through acquired temporal associations with the traumatic event [57]. This conditioned threat to sensory cues one encounters in everyday life may lead to exaggerated externally-oriented attention to detect and respond to such threat cues—the hallmark presentation of hypervigilance. Translational evidence suggests that “hypervigilance” and its proposed neurochemical underpinnings increase sensory sensitivity, reduce sensory gating, and facilitate rapid sensory processing, potentially through alterations in attention [58–67]. Therefore, hypervigilance may contribute to the detection and rapid processing of sensory cues that trigger intrusive memories and flashbacks through the activation of sensory-bound representations of the traumatic event. Moreover, physiological arousal may further exacerbate this cycle, as inductions of physiological arousal can elicit flashback-like memories [68–70]. Taken together, these findings suggest that the IH phenotype represents a clinical presentation of posttraumatic stress associated with interactions between arousal, memory, and attention systems that amplify specific reexperiencing symptoms.

In keeping with this notion, the observed elevations in circulating PACAP levels within the IH phenotype align with the demonstrated role of PACAP in stress physiology and fear memory. PACAP is a critical regulator of arousal through its signaling in autonomic stress pathways [33, 34, 71]. PACAP and its predominant type 1 receptor (PAC1R) are densely expressed within the central extended amygdala [32–35], encompassing both the central amygdala (CeA) and bed nucleus of the stria terminalis (BNST), which activate fear and panic reflexes in response to threat through downstream projections to physiological arousal systems [72]. PACAP is also linked to the acquisition, consolidation, and extinction of contextual or cue-based fear memory through alterations in synaptic plasticity within fear memory circuits [73–77]. Notably, PACAP signaling facilitates trace fear memory, a form of fear learning that is dependent on sustained attention and episodic memory systems [78] and is highly aligned with the “warning signal” model of intrusive reexperiencing symptoms. To this end, PACAP may facilitate attentional states of hypervigilance for threat as a consequence of sustained conditioned fear. Moreover, PACAP may enhance consolidation and impair extinction of contextual fear memories that are linked to episodic memory systems, akin to trauma-related intrusive memories and flashbacks. Aligning with the neurocognitive models of PTSD, this cycle may be maintained and exacerbated by the elevated states of physiological arousal driven by PACAP signaling. Therefore, PACAP is optimally positioned at the intersection of arousal, memory, and attention systems to give rise to the IH phenotype.

Strengthened amygdala FC with the identified posterior and prefrontal regions further supports a role of arousal-memory-attention interactions. Stronger functional coupling between the amygdala and precuneus is associated with greater threat reactivity [79], hyperarousal [42], and sustained attention to aversive stimuli [80]. Additionally, both the amygdala and our identified precuneus cluster are linked to the retrieval of emotional autobiographical memory [81–84]. Our identified mPFC cluster overlaps with the pregenual ACC (Brodmann’s Area 32), whose rodent analogue, the prelimbic cortex, is consistently implicated in the expression of conditioned fear through connectivity with the amygdala [85–87]. Specifically, the retrieval of long-term conditioned fear memory may be supported by strengthened connections between the prelimbic cortex and central amygdala through the paraventricular thalamus [88, 89]. In humans, the pregenual ACC is engaged during the retrieval of remote contextual fear memories that involve the amygdala and hippocampus [90–93]. Similarly, the POS and adjacent visual cortex have been tied to the retrieval of vivid sensory details of episodic memories [31, 94–96] through their role in visual imagery and visuospatial processing [97, 98]. More broadly, these posterior cortices are involved in emotional arousal [99], vigilance [100], and directed attention [101]. In sum, while these regions support numerous functions, they converge on a pattern of processes that are tied to the detection, processing, and responding to threat and the consolidation and retrieval of emotional episodic or fear memories. Our meta-analytic decoding of this collective pattern of posterior and prefrontal clusters provides evidence for this convergence, as it revealed a spatial overlap with studies ascribing functions such as mental imagery, memory retrieval and encoding, negative emotions, fear and threat, and attention (Supplementary Table 4).

Collectively, these identified regions fall predominantly within the DMN and Visual Network (Fig. 3B). Both networks are consistently implicated in posttraumatic stress given their respective functions in episodic memory, attention, and arousal [36, 102–107]. Typically, these networks demonstrate robust anticorrelation and are positioned at opposite ends of the hierarchical organization of cortical networks, reflecting a maximal segregation in their patterns of activity and connectivity [53]. However, there is accumulating evidence for a breakdown in the functional segregation of these intrinsic networks in posttraumatic stress [108, 109], particularly in relation to symptoms of hypervigilance [104] and trauma reexperiencing [110]. Additionally, an imbalance in their anticorrelation, or co-deactivation, has been linked to the sensory-perceptual features of trauma memories, including their emotional intensity and sense of reliving, which are key features of flashbacks [95]. The current findings of strengthened connectivity between the amygdala and central brain regions of both the DMN and Visual Network may similarly reflect increased functional integration of these normally divergent networks. This suggests that the IH clinical phenotype is marked by abnormal coupling between these intrinsic resting state networks and the amygdala, reflecting a potential biomarker for this subtype. Future work incorporating dynamic FC of these networks is needed to elucidate the network-wide versus regionspecific alterations and which patterns maximally differentiate the IH phenotype. Moreover, task-based neuroimaging may be able to determine whether these changes in organization are in fact tied to behavioral measures of arousal, memory, and attention.

Interestingly, our amygdala FC findings were specific to the rCMA, as no effects emerged with the lCMA or r/lBLA. The BLA generally demonstrates more robust FC with cortical structures compared to the CMA, which preferentially connects with subcortical structures like the midbrain and striatum [111–114]. Our findings replicate this pattern, as the non-IH groups demonstrated near-zero average connectivity between the CMA and cortical regions (Fig. 3C). Prior work has demonstrated increased BLA-cortical connectivity in PTSD compared to trauma-exposed controls, but no differences in CMA connectivity due potentially to the minimal intrinsic CMA-cortical connectivity [111]. However, we previously demonstrated that elevated levels of circulating PACAP are associated with strengthened cortical connectivity of the rCMA, and not the BLA, with midline regions of the DMN, including the precuneus [42]. Our present findings qualify this unusual strengthening of CMA-cortical connectivity by demonstrating a specificity of this effect to the IH clinical phenotype, which was the only clinical phenotype with elevations in PACAP levels. Therefore, this abnormal rCMA connectivity pattern may reflect a PACAP-mediated shift in the functional organization of the amygdala and its cortical interactions that is specific to the IH phenotype. These converging findings position the combination of high PACAP and rCMA FC as a candidate biomarker for the IH clinical phenotype, reflecting a distinct neurobiological profile not observed in other posttraumatic stress presentations.

Critically, the High and Low symptom groups did not differ in PACAP or rCMA FC despite notable differences in nearly every symptom, including intrusive memories and hypervigilance (Fig. 1). This is consistent with Adams et al. [16], who likewise reported nearly identical PACAP levels between these two groups despite notable differences in nearly every PTSD symptom. This specificity reinforces the hypothesis that the IH phenotype may reflect a biologically distinct subtype of posttraumatic stress, characterized clinically by differentially elevated intrusive memories, flashbacks, and hypervigilance. Among these, flashbacks emerged as the only individual symptom significantly associated with PACAP and rCMA connectivity. This may be attributable to the greater physiological arousal and cognitive alterations often associated with flashbacks compared to other trauma-related intrusive memories, reflecting an exaggerated interaction among memory, attention, and arousal systems [115]. Neuroimaging studies examining the reliving or re-enacting of traumatic events further emphasize a coupling between the DMN and sensory cortex in flashbacks, reflecting a reorganization of intrinsic connectivity patterns that is marked by the integration of sensory processing and autobiographical memory retrieval [108]. Notably, flashbacks were also the most robust differentiator between the IH and non-IH groups. These findings suggest that flashbacks may serve as a core clinical marker of the distinct biological profile underlying the IH phenotype. Because flashbacks are characterized by vivid, sensory-based reliving of the traumatic event, this profile may be particularly amenable to precision therapeutics that target the sensory elements of posttraumatic reexperiencing [61, 110, 116], either through neurofeedback [117], brain stimulation [118–120], or more integrative approaches [121]. Although speculative, these possibilities highlight how delineating biologically-informed PTSD subtypes may ultimately guide personalized treatment development. External replications with independent trauma-exposed samples are needed to confirm this specificity and validate these biomarkers of an IH phenotype, including the potential centrality of flashback symptoms.

The present findings are not without limitations. First, although we replicated prior findings of elevated PACAP in the IH phenotype, the effect sizes were markedly smaller than those reported by Adams et al.[16]. Several factors may have contributed to these discrepancies. Notably, the present study used a single measurement of PACAP, whereas Adams et al. acquired three consecutive measurements, which may yield more reliable estimates of circulating PACAP levels. Additionally, Adams et al. had a more even distribution of male and female participants as well as a more diverse sample with regard to ethnoracial identity. They further demonstrated that the IH phenotype was characterized by a higher proportion of women from marginalized ethnoracial groups; therefore, our predominantly white female sample may have lacked the sociocultural and stress-related diversity to capture these nuanced effects. More broadly, variability in PACAP levels may reflect heterogeneity in trauma exposures, including cumulative stressors faced by women from marginalized ethnoracial groups. Thus, future studies incorporating repeated PACAP assessments and more diverse samples, as well as comprehensively characterizing trauma history and chronic stress burden, will be critical for clarifying the conditions under which PACAP most robustly indexes the IH phenotype. As this study represents the first independent replication of the IH phenotype, any proposed guidelines for its derivation are necessarily preliminary. Nonetheless, the convergence of findings across latent class and latent profile approaches suggests that the IH clinical presentation phenotype represents a reproducible pattern of elevated intrusive reexperiencing and hyperarousal symptoms that is not dependent on the specific clustering method. Future studies should examine the stability of this phenotype across and within subjects, and determine whether empirically derived symptom thresholds can support clinically meaningful identification.

Additional limitations include the cross-sectional design of our study, which precludes inference of directionality in associations between PACAP, rCMA connectivity, and clinical phenotypes. Longitudinal studies are needed to gain insights into the temporal relationship and interdependency between these biological and clinical markers. Relatedly, all imaging data were collected at rest; as such, we cannot directly support claims linking PACAP and rCMA-cortical connectivity to the active processes of memory, attention, and arousal that are implicated in intrusive reexperiencing and hypervigilance symptoms. Future studies incorporating cognitive testing or behavioral measures of these constructs in conjunction with task-based neuroimaging are needed to clarify the mechanistic processes giving rise to the IH phenotype. Additionally, prior work has demonstrated interactions between PACAP and estrogen [40, 122]. While our current sample did not differ with regard to menstrual status, hormonal contraceptive use, or history of hysterectomy across the identified clinical phenotypes (Table 1), more nuanced investigations into the interaction between estrogen and PACAP are encouraged in order to provide critical insights into the role of biological sex in the IH phenotype. Finally, our FC analyses were constrained to amygdala subregions, specifically the BLA and CMA, given prior work demonstrating robust PACAP signaling within these regions. However, PACAP is expressed in numerous other subcortical and midbrain regions that are tied to behavioral arousal, attention, and memory, such as locus coeruleus (LC) and the bed nucleus of the stria terminalis (BNST) [33]. Many of these structures require greater anatomical precision or individual-level, hand-drawn anatomical masks to delineate them from adjacent structures and sources of physiological noise. Future studies utilizing ultra-high-field MRI (i.e., 7 T) or specialized sequences (e.g., neuromelanin-sensitive imaging) would afford deeper insights into other subcortical structures that may be mechanistically linked to the IH phenotype.

In conclusion, this study replicates the identification of an intrusive-hypervigilant clinical phenotype of posttraumatic stress that is characterized by elevated circulating PACAP levels and provides novel evidence for altered patterns of FC of the rCMA. This IH phenotype may reflect a distinct clinical presentation that is characterized by exaggerated interactions between memory and arousal processes, thus driving specific symptoms that are situated at this intersection, like flashbacks. Our findings further add to the accumulating evidence linking both PACAP and altered organization of subcortical-cortical networks to memory, arousal, and PTSD. More specifically, the IH phenotype exhibits these biomarkers previously implicated in the PTSD literature, which have been stymied by inconsistent replication across different, diverse samples. Therefore, the IH phenotype may be a candidate clinical presentation for a precision psychiatry framework that targets these biomarkers through pharmacotherapy or neurotherapeutics.

## Supplementary Material

Supplement

ADDITIONAL INFORMATION

Supplementary information available at https://doi.org/10.1038/s41386-026-02396-0.

## Figures and Tables

**Figure 1.**
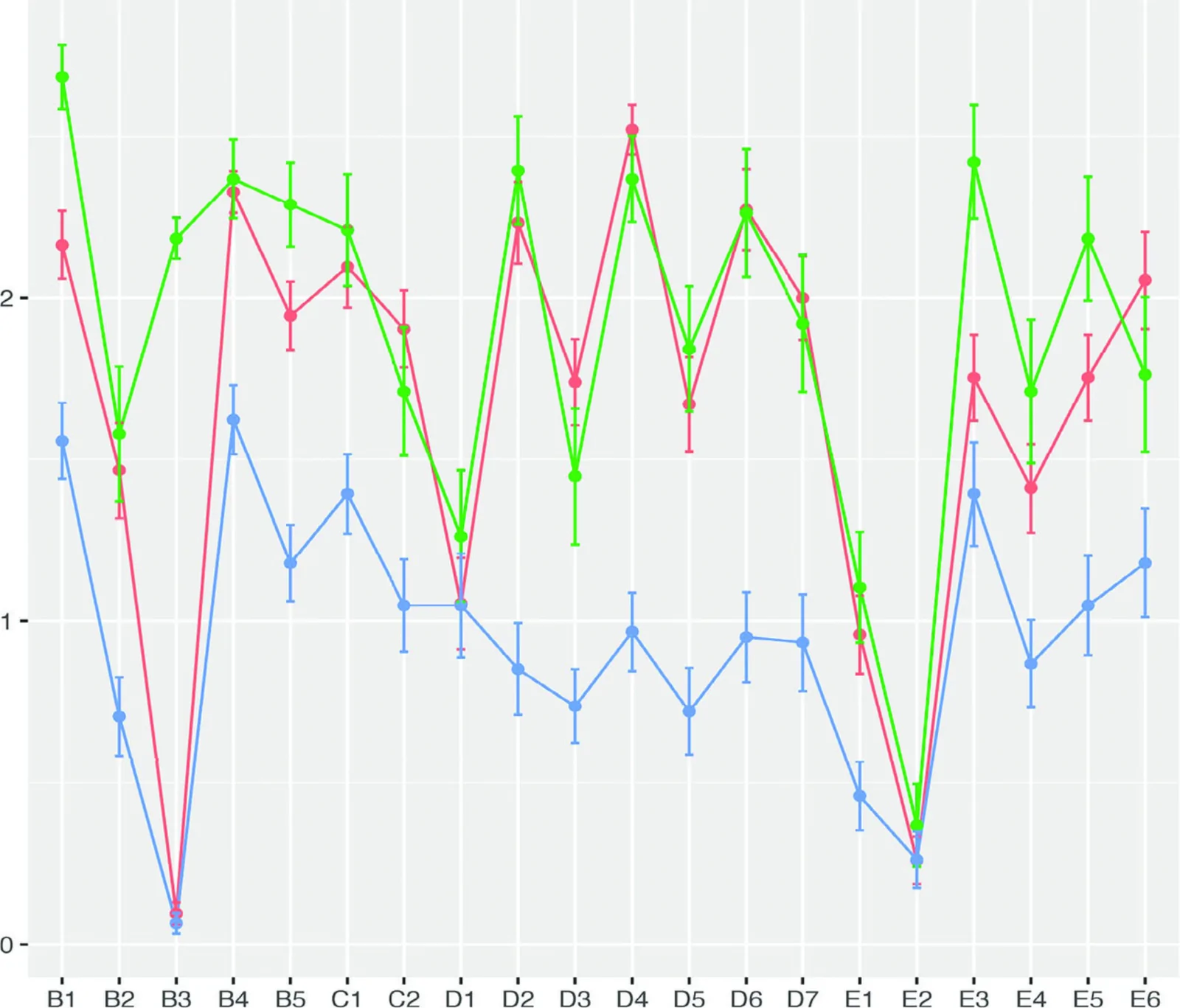
CAPS-5 symptom profiles of the groups identified from the latent profile analysis. * indicates where the Intrusive-Hypervigilant (IH) group differed from both the High and Low groups. B = Intrusive Reexperiencing symptoms (Cluster B); C = Avoidance symptoms (Cluster C); D = Negative Alterations in Cognitions and Mood symptoms (Cluster D); E = Hyperarousal symptoms (Cluster E).

**Figure 2.**
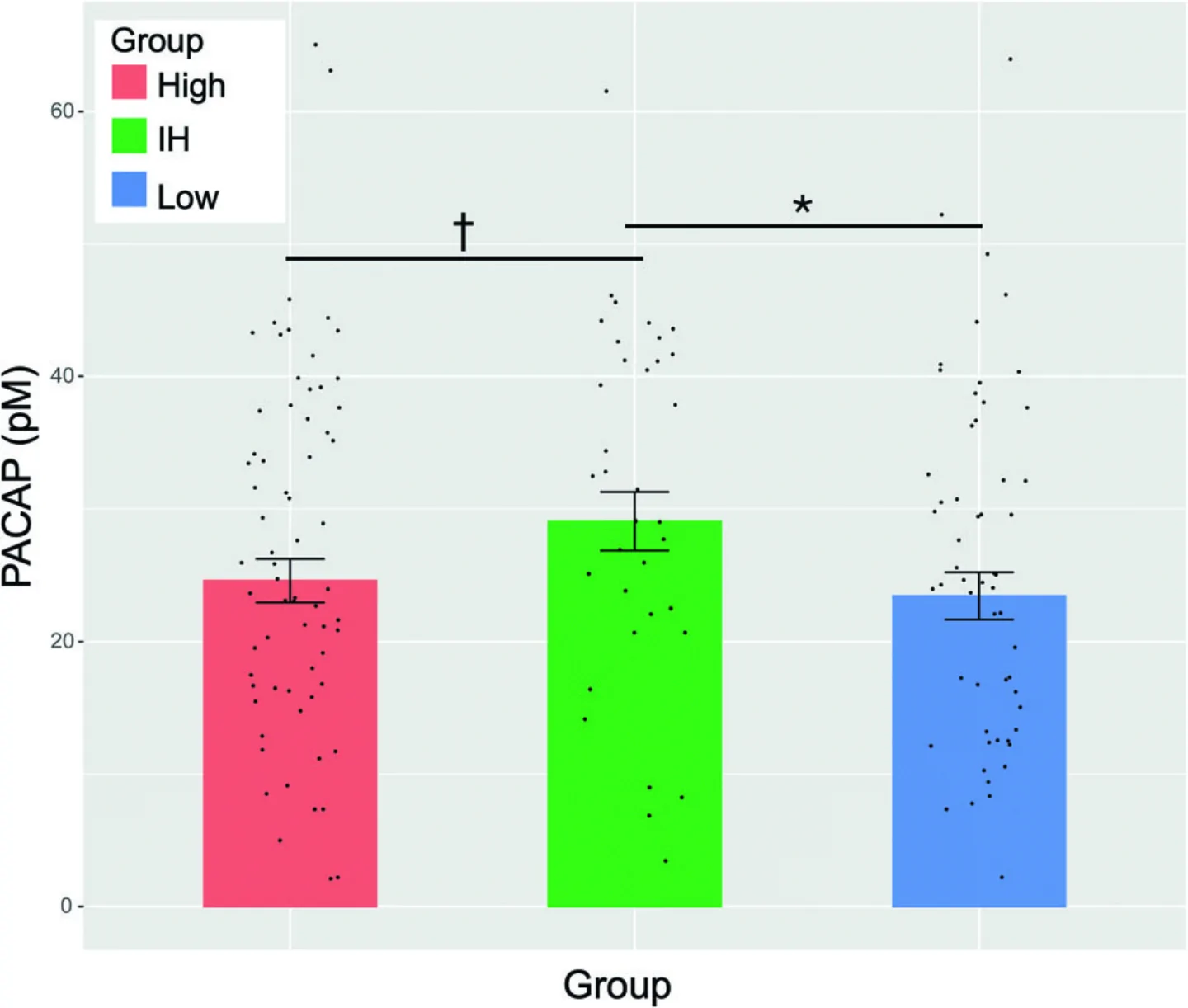
Differences in circulating PACAP levels across symptom subtypes. Participants with selectively elevated Intrusive-Hypervigilant (IH) PTSD symptom profiles demonstrated greater PACAP levels than participants with generally high (High) and generally low (Low) PTSD symptom profiles. Bars represent estimated marginal means from linear regression models controlling for batch effects; error bars reflect standard errors from the mean. Individual points reflect raw (untransformed) PACAP values. PACAP values were square-root transformed for statistical analysis. † p = 0.05 one-tailed; * p < 0.05 one-tailed.

**Figure 3.**
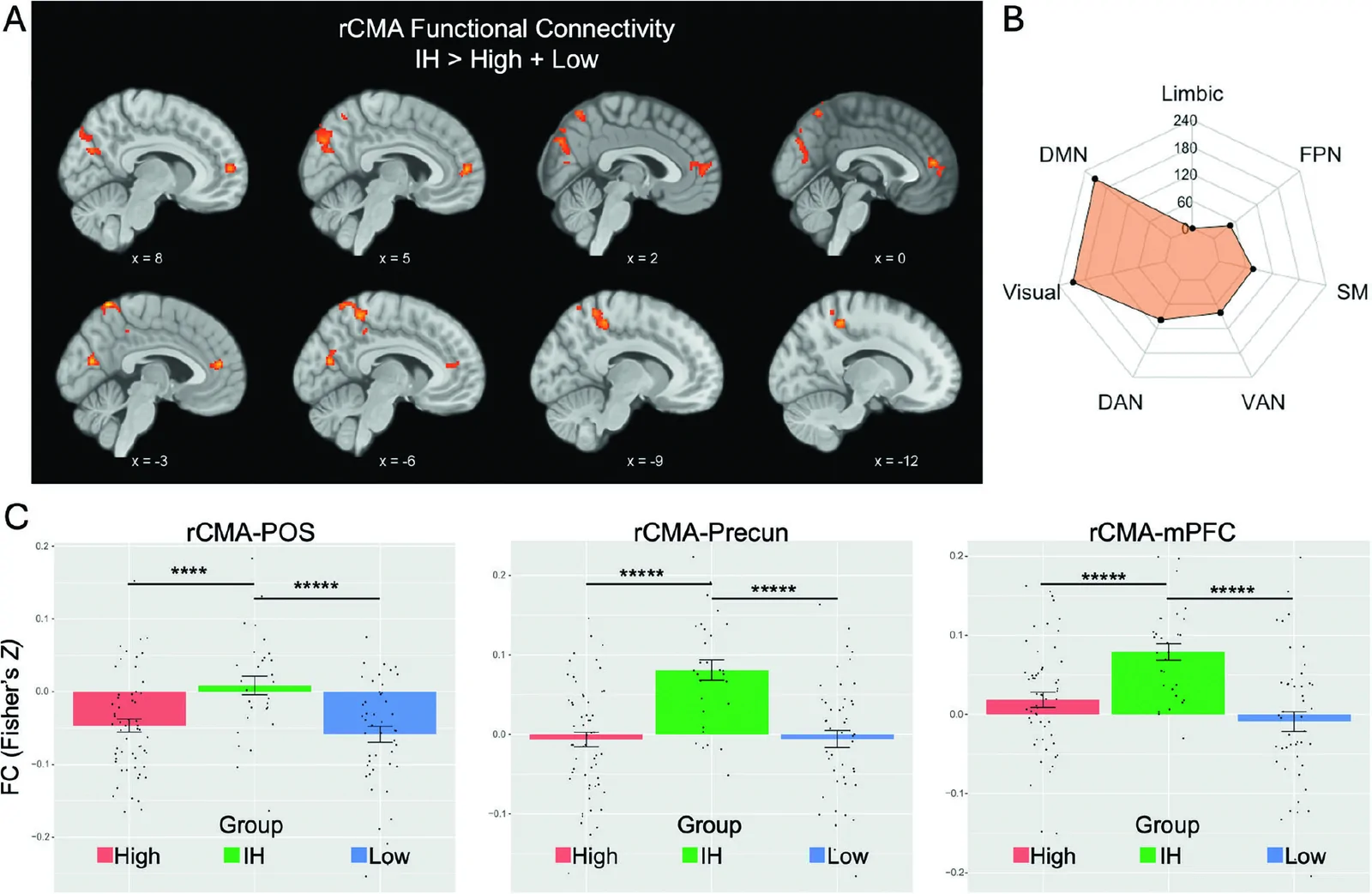
Group differences in right centromedial amygdala (rCMA) resting-state functional connectivity (FC). A Whole-brain contrasts (IH > non-IH) revealed three significant clusters showing greater rCMA FC in the Intrusive-Hypervigilant (IH) group within the parietooccipital sulcus (POS), precuneus (Precun), and medial prefrontal cortex (mPFC). B Radar plot depicting the number of voxels within the significant clusters that overlap with the 7 networks of the Yeo atlas, demonstrating the clusters were predominantly within the default mode network (DMN) and visual network. C Post hoc individual group contrasts revealed that the IH group showed significantly greater rCMA FC than both the High and Low symptom groups across all three clusters. FPN frontoparietal network, SM somatomotor network, VAN ventral attention network, DAN dorsal attention network, **** < 0.001, ***** < 0.0005, ****** < 0.0001.

**Figure 4.**
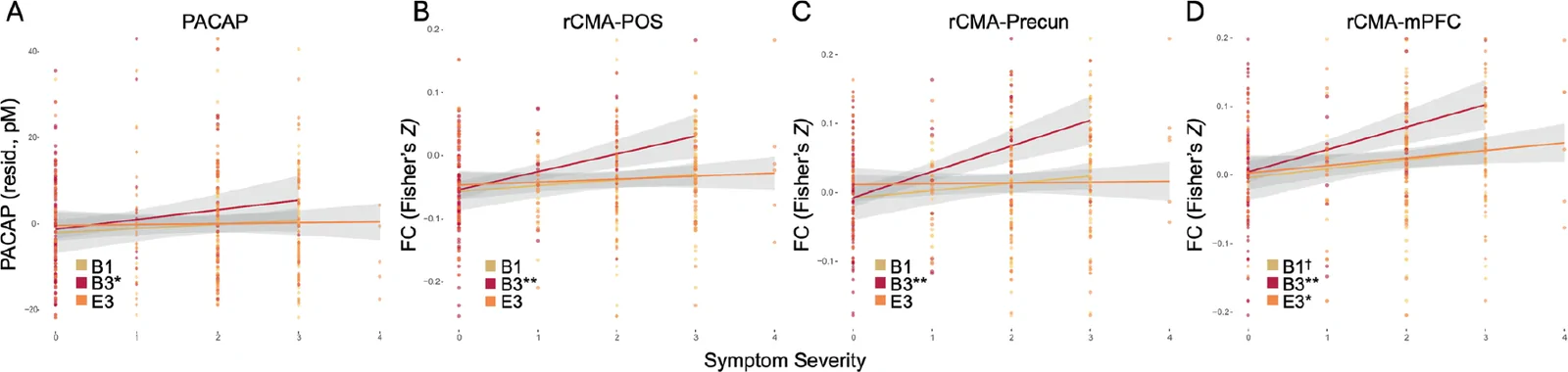
Associations between posttraumatic stress disorder (PTSD) symptom severity and biomarkers of interest. A Pituitary adenylate cyclase-activating (PACAP) levels (square-root-transformed) were significantly associated with flashback severity (CAPS-5, item B3) but not with trauma-related intrusive memories (B1) or hypervigilance (E3). B-D Right centromedial amygdala (rCMA) FC with the parieto-occipital sulcus (POS), precuneus (Precun), and medial prefrontal cortex (mPFC) was positively associated with flashback severity. Weaker, trending associations were also observed between rCMA-mPFC connectivity and both intrusive memories and hypervigilance. CAPS-5: Clinician-Administered PTSD Scale for DSM-5.

**Table 1.** Demographic and clinical characteristics of the sample.

Characteristics	Total (N = 172)	IH Group (n = 38)	High Group (n = 73)	Low Group (n = 61)	Group Difference
Age (years)	30.3 ± 8.9	29.7 ± 8.4	31.1 ± 9.5	29.7 ± 8.5	n.s.
**Racial Identity (%)**					n.s
Asian	15 (9%)	4 (11%)	5 (7%)	6 (10%)	
Black	17 (10%)	4 (11%)	8 (11%)	5 (8%)	
Native American or Alaskan Native	2 (1%)	0	1 (1%)	1 (2%)	
White	109 (63%)	21 (55%)	49 (67%)	39 (64%)	n.s
Bi-/multiracial	17 (10%)	6 (16%)	7 (10%)	4 (7%)	
Other or Not Reported	12 (7%)	3 (8%)	3 (4%)	6 (10%)	
**Ethnicity (%)**					
Hispanic	31 (18%)	7 (18%)	13 (18%)	11 (18%)	n.s.
Non-Hispanic	141 (82%)	31 (82%)	60 (82%)	50 (82%)	
**Gender (%)**					
Woman	102 (59%)	28 (74%)	43 (59%)	31 (51%)	IH > High, Low
Man	60 (35%)	8 (21%)	24 (33%)	28 (46%)	
Non-binary or gender non-conforming	10 (6%)	2 (5%)	6 (8%)	2 (3%)	
**Medication (%)**					n.s.
SSRI	36 (21%)	8 (21%)	17 (23%)	11 (18%)	
Mood Stabilizer	16 (9%)	5 (13%)	5 (7%)	6 (10%)	
Antipsychotic	1 (1%)	1 (3%)	0	0	
Benzodiazepine	9 (5%)	3 (8%)	3 (4%)	3 (5%)	
SNRI	9 (5%)	4 (11%)	2 (3%)	3 (5%)	
Stimulant	12 (7%)	4 (11%)	5 (7%)	3 (5%)	
Anti-hypertensive	4 (2%)	1 (3%)	0	3 (5%)	
Atypical Antidepressant	27 (16%)	8 (21%)	12 (16%)	7 (11%)	
Anxiolytic	25 (15%)	7 (18%)	11 (15%)	7 (11%)	
Sex Assigned at Birth (female/ male)	108/64	30/8	46/27	32/29	IH > High, Low
Currently menstruating* (yes/no)	85/20	21/7	40/6	24/7	n.s.
Regular Menstrual Cycles* (yes/no)	72/28	16/9	36/9	20/10	n.s.
Current Birth Control* (yes/no)	42/63	10/18	17/29	15/16	n.s.
Hysterectomy* (yes/no)	4/100	0/28	2/43	2/29	n.s.
PTSD Diagnosis (%)	115 (67%)	30 (79%)	63 (86%)	22 (36%)	IH, High > Low
CAPS Total	29.4 ± 10.4	38.1 ± 9.4	33.7 ± 6.0	19.0 ± 5.4	IH > High > Low
TLEQ Total Number of Events	24.8 ± 15.8	26.9 ± 16.7	25.0 ± 14.8	22.0 ± 15.2	n.s.

Sex assigned at birth, gender identity, race, and ethnicity data were acquired via participant self-report. IH Intrusive-Hypervigilant, CAPS-5 Clinician Administered PTSD Scale for DSM-5, TLEQ Traumatic Life Events Questionnaire [123]. Means ± standard deviations or N (%).

## Data Availability

Data are available through the NIMH National Data Archive (NDA; https://nda.nih.gov/edit_collection.html?id=3166) and are available upon reasonable request to the senior author, IMR.
