# Supplementary material for: Identification of an intrusive-hypervigilant phenotype of posttraumatic stress symptoms with unique stress peptide and amygdala functional connectivity profiles
Source: Neuropsychopharmacology. Author manuscript; Available in PMC 2026 Sep 29. (PMC13598154; doi:10.1038/s41386-026-02396-0)
Supplement: Supplementary file 1 [file NIHMS2207593-supplement-Supplement.docx]

**SUPPLEMENTAL MATERIALS**

*Identification of an intrusive-hypervigilant phenotype of posttraumatic stress symptoms with unique stress peptide and amygdala functional connectivity profiles – Clancy et al.*

**METHODS**

***PACAP***

PACAP38-specific immunoassays (Cat. No. HUFI02692, AssayGenie, Dublin, Ireland) were performed at the University of Vermont - Larner College of Medicine using human plasma samples that were collected at the beginning of each study visit, between approximately 8:00-10:00AM. Samples were centrifuged at 3500 rpm for 15 minutes. Plasma was extracted and stored at -80 °C until analysis. Optimal sample volume was determined in dilution tests, and all values represent the mean from assay duplicates; intra-assay variation was approximately 9%. Assay midpoint was 1.1 fmol and detection limit from the linear range of the standard curve was 0.2 fmol.

***Latent Symptom Profile Analysis***

Four models with different covariance structures were examined, each testing a range of 1 to 4 profiles (as in, Adams et al. 2025), resulting in a total of 16 models examined. In two of the models, clustered profiles were assumed to have equal volume and shape, with one model having an equal diagonal covariance matrix (EEI model) and the other allowing for full covariance matrices (EEE model) across profiles. The other two models permitted variable volumes and shapes across profiles, with one utilizing an equal diagonal covariance matrix (VVI model) and the other allowing full covariance matrices (VVV model) across profiles. Optimal model fit was determined using Bayesian Information Criterion (BIC), comparing BIC values for all 16 different model fits. Descriptive statistics of all 20 CAPS-5 items for each identified clinical profiles (i.e., groups) are reported in **Supplemental Table 2**.

***MRI data acquisition and preprocessing***

Imaging was conducted at the McLean Hospital Imaging Center on a 3T Siemens Prisma scanner with a 64-channel head coil using Human Connectome Project (HCP) sequences for anatomical and functional images. MRI data were preprocessed using fMRIPrep version 20.2.7 [1] with non-aggressive AROMA denoising of motion artifacts [2]. Participants were excluded if their mean framewise displacement (FD) exceeded 0.5mm or greater than 20% of volumes exceeded FD = 0.5 mm (n = 14). Subsequent denoising of residual physiological confounds from white matter and cerebrospinal fluid using the anatomical CompCor method, scrubbing of motion outliers (FD > 0.5mm), and high pass (0.1 Hz) filtering were performed using CONN [3]. Importantly, aCompCor components were estimated in CONN from the AROMA-cleaned data (i.e., after non-aggressive AROMA denoising) using white matter and cerebrospinal fluid anatomical masks generated by fMRIPrep. This approach was chosen instead of using the aCompCor components estimated by fMRIPrep, which estimates aCompCor components prior to AROMA denoising, to avoid reintroducing noise while accounting for any remaining physiological noise not captured by AROMA motion-related components. In addition to the 14 excluded from analysis for excessive motion, 7 had incomplete scanning, 4 had inadequate alignment of structural and functional scans, 6 had significant imaging artifact, and 7 had no scanning performed, resulting in a final sample of n = 136 for fMRI analyses.

***Analytic Plan***

*Power Analysis*

Based on the original manuscript identifying the IH phenotype [4], the smallest PACAP effect size they observed was η_p_^2^=.52, necessitating a sample size of N = 39 with an alpha = 0.05 and Power set to 0.8.

*Bayesian Statistics*

To complement our frequentist approach for PACAP analyses in the main manuscript, which was chosen for the sake of consistency with the initial study identifying the IH phenotype [4], we conducted Bayesian linear regression using the BayesFactor package in R. We compared a model including clinical phenotype (IH vs. non-IH) group as a predictor of PACAP, controlling for Batch, against a model including Batch alone. This model comparison yields a Bayes factor (BF₁₀) representing the relative evidence for the inclusion of clinical phenotype group status over and above batch effects. Default priors were used for regression coefficients. This approach allows direct quantification of evidence for both the presence and absence of group differences while accounting for batch-related variance.

**RESULTS**

***Bayes factor for PACAP findings***

Bayesian model comparison showed minimal or anecdotal evidence for the IH group exhibiting greater PACAP levels compared to the non-IH group (BF₁₀ = 1.23) when accounting for potential batch effects.

***Mixed Effects Model for PACAP findings***

A mixed effects model examining the fixed effect of IH Group on PACAP levels with Batch as a random effect confirmed an effect of IH Group (IH > non-IH; B = 0.37, *t* = 2.04, *p* = 0.043). Batch-stratified models revealed a robust effect of IH Group in Batch 2 (t = 2.66, p = 0.009) but not Batch 1 (t = 0.73, p = 0.464).

***Associations of Sex Assigned at Birth***

There was a robust association between biological sex and PACAP levels (females > males; t = 5.08, p < 0.001). When incorporating sex as a covariate in models examining Group (IH vs. non-IH) difference in PACAP levels while controlling for Batch, the main effect of Group was no longer significant (t = 1.12, p = 0.264), consistent with the magnitude of sex-related variance. There was no evidence of a Sex Group interaction in relation to PACAP levels (p = 0.902), and sex-stratified analyses likewise did not indicate Group differences within females (IH vs. non-IH; t = 0.97, p = 0.335) or males (IH vs. non-IH; t = 0.54, p = 0.593). However, given the relatively modest sample size and uneven sex distribution, these sex-stratified analyses should be interpreted with caution (Supplemental Table 5).

For functional connectivity, sex assigned at birth was associated with rCMA-POS FC (female > males; t = 2.92, p = 0.004) but not rCMA-Precun or mPFC FC (p’s > 0.4202). When sex was included as a factor in models relating rCMA FC to Group, the Group differences remained significant for rCMA-POS (F = 17.56, p = < 0.001), rCMA-Precuneus (F = 33.89, p < 0.001), and rCMA-mPFC FC (F = 21.55, p < 0.001), with no Sex x Group interactions (p’s > 0.765). Sex-stratified analyses similarly demonstrated significant Group differences in both male and female participants (Supplemental Table 5). Therefore, in contrast to PACAP levels, the IH-related CMA connectivity differences persist above and beyond the influence of sex.

***Associations of PTSD diagnosis or total PTSD severity***

There was no association of PTSD diagnosis (threshold vs. subthreshold PTSD) with PACAP levels when controlling for Batch (t = -0.149, p = 0.882). Similarly, there was no association between total PTSD severity (total CAPS-5 score) and PACAP levels when controlling for Batch (B = 0.12, p = 0.130). The group differences in PACAP persisted when including PTSD diagnosis as a covariate (IH > non-IH; t = 1.98, p = 0.049), and there was no interaction of PTSD diagnosis x Group on PACAP (p = 0.808). However, the IH group association with PACAP was no longer significant when controlling for total PTSD severity (t = 1.46, p = 0.145), with no interaction effect (p = 0.342).

There were no associations of PTSD diagnosis with rCMA FC (p’s > 0.251). There were weak associations between CAPS severity and rCMA-POS (r = 0.20, p = 0.022) and rCMA-mPFC FC (r = 0.21, p = 0.013), but not rCMA-Precuneus FC (r = 0.09, p = 0.278). The IH group associations with rCMA FC persisted when including PTSD diagnosis as a covariate (F’s > 16.91, p’s < 0.001), with no interaction effects (p’s > 0.232). The associations of IH group with rCMA FC remained significant when including CAPS severity as a confounding covariate (F’s > 16.89, p’s < 0.001), and there were no interaction effects (p’s > 0.466).

***Associations with medication status***

Individuals who were taking prescribed psychotropic medication (n = 82) did not differ from those not taking psychotropic medication (n = 90) in PACAP levels (p = 0.133) or rCMA FC (p’s > 0.050). As such, group differences (IH vs. non-IH group) on PACAP levels remained when controlling for medication status (t = 1.84, p = 0.068, p = 0.034 one-tailed). Similarly, the association of IH group (vs. non-IH group) with rCMA FC persisted when controlling for medication status (rCMA-POS: t = 3.90, p = 0.0002; rCMA-Precun: t = 5.66, p < 0.0001; rCMA-mPFC: t = 4.51, p < 0.0001).

Furthermore, excluding participants who were taking prescribed benzodiazepines or stimulants (n = 21) revealed stronger group differences in PACAP levels (IH > non-IH; t = 2.16, p = 0.032, p = 0.016 one-tailed) and rCMA FC (rCMA-POS: t = 4.13, p = 0.0001; rCMA-Precun: t = 5.94, p < 0.000001; rCMA-mPFC: t = 5.52, p < 0.000001).

***Associations with trauma chronicity***

Trauma chronicity, as indexed by the total number of different traumatic events indicated on the TLEQ, was not associated with PACAP levels (p = 0.122) or rCMA FC (p’s > 0.339). As such, the group differences (IH vs. non-IH) on PACAP levels persisted when controlling for trauma chronicity (t = 1.96, p = 0.052, p = 0.026 one-tailed). Similarly, the association of IH group (vs. non-IH group) on rCMA FC persisted when controlling for trauma chronicity (rCMA-POS: t = 4.13, p = 0.0001; rCMA-Precun: t = 5.91, p < 0.0001; rCMA-mPFC: t = 4.66, p < 0.0001).

***Associations with dissociative symptoms***

Given the prominent role of dissociation in flashbacks, we examined the association of CAPS-5 dissociative symptoms (total score summing depersonalization and derealization items) with PACAP and functional connectivity. Across all participants, PACAP levels were modestly associated with CAPS-5 dissociative symptoms (β = 0.17, SE = 0.09, *p* = 0.073). Dissociative symptoms were additionally associated with rCMA-POS (r = 0.28, p = 0.001) and rCMA-mPFC (r = 0.18, p = 0.037) FC, but not rCMA-Precun FC (r = 0.12, p = 0.185).

**SUPPLEMENTAL TABLES**

**Supplemental Table 1.** Comparison of participants who were included vs. excluded from fMRI analyses.

|  | Included vs. Excluded |
| --- | --- |
| Sex at Birth (female/male) | χ^2^ = 0.16, p = 0.689 |
| PACAP | t = 0.70, p = 0.482 |
| CAPS-5 Total Score | t = 0.18, p = 0.862 |
| CAPS-5 B1 | t = 0.70, p = 0.488 |
| CAPS-5 B3 | t = -0.70, p = 0.489 |
| CAPS-5 E3 | t = 0.03, p = 0.974 |
| PTSD Diagnosis | χ^2^ = 0.34, p = 0.558 |

**Supplemental Table 2.** Means and Standard Deviations of each CAPS-5 symptom across the 3 clinical groups.

| **Symptom** | **High Group** Mean (SD) | **IH Group**  Mean (SD) | **Low Group**  Mean (SD) |
| --- | --- | --- | --- |
| B1 | 2.16 (0.90) | 2.68 (0.62) | 1.56 (0.92) |
| B2 | 1.47 (1.26) | 1.58 (1.29) | 0.70 (0.95) |
| B3 | 0.10 (0.30) | 2.18 (0.39) | 0.07 (0.25) |
| B4 | 2.33 (0.55) | 2.37 (0.75) | 1.62 (0.84) |
| B5 | 1.95 (0.90) | 2.29 (0.80) | 1.18 (0.92) |
| C1 | 2.10 (1.07) | 2.21 (1.07) | 1.39 (0.95) |
| C2 | 1.90 (1.02) | 1.71 (1.23) | 1.05 (1.12) |
| D1 | 1.05 (1.21) | 1.26 (1.25) | 1.05 (1.26) |
| D2 | 2.23 (1.09) | 2.39 (1.03) | 0.85 (1.11) |
| D3 | 1.74 (1.14) | 1.45 (1.29) | 0.74 (0.89) |
| D4 | 2.52 (0.65) | 2.37 (0.82) | 0.97 (0.95) |
| D5 | 1.67 (1.25) | 1.84 (1.20) | 0.72 (1.05) |
| D6 | 2.27 (1.07) | 2.26 (1.22) | 0.95 (1.09) |
| D7 | 2.00 (1.11) | 1.92 (1.30) | 0.93 (1.17) |
| E1 | 0.96 (1.03) | 1.11 (1.06) | 0.46 (0.83) |
| E2 | 0.26 (0.62) | 0.37 (0.79) | 0.26 (0.68) |
| E3 | 1.75 (1.14) | 2.42 (1.08) | 1.39 (1.24) |
| E4 | 1.41 (1.16) | 1.71 (1.37) | 0.87 (1.06) |
| E5 | 1.75 (1.14) | 2.18 (1.18) | 1.05 (1.20) |
| E6 | 2.05 (1.28) | 1.76 (1.48) | 1.18 (1.31) |

**Supplemental Table 3.** Individual CAPS-5 symptom group contrasts of resting-state rCMA FC.

|  | IH vs. High | IH vs. Low | High vs. Low |
| --- | --- | --- | --- |
| rCMA-POS | *t* = 3.43, *p* = 0.0008, d = 0.78 | *t* = 4.05, *p* = 0.0001, d = 0.95 | *t* = 0.87, *p* = 0.385, d = 0.20 |
| rCMA-Precuneus | *t* = 5.38, *p* < 0.0001, d = 1.21 | *t* = 5.15, *p* < 0.0001, d = 1.20 | *t* = 0.05, *p* = 0.960, d = 0.01 |
| rCMA-mPFC | *t* = 3.60, *p* = 0.0004, d = 0.81 | *t* = 5.04, *p* < 0.0001, d = 1.18 | *t* = 1.85, *p* = 0.067, d = 0.35 |

CAPS-5 = Clinician-Administered PTSD Scale for DSM-5; IH = Intrusive-Hypervigilant; rCMA = right centromedial amgydala, POS = parietooccipital sulcus, mPFC = medial prefrontal cortex.

**Supplemental Table 4.** Meta-analytic decoding of significant FC clusters.

| **NeuroSynth Meta-Analysis Term** | **Z-Score** |
| --- | --- |
| 0_network_state_resting | 3.33 |
| 8_mpfc_social_medial | 2.63 |
| 41_imagery_mental_events | 1.74 |
| 3_cortex_anterior_cingulate | 1.55 |
| 33_memory_retrieval_encoding | 1.50 |
| 30_decision_making_risk | 0.92 |
| 26_emotional_amygdala_negative | 0.90 |
| 24_age_adults_older | 0.89 |
| 28_social_empathy_moral | 0.74 |
| 48_prefrontal_cortex_pfc | 0.70 |
| 7_reward_feedback_striatum | 0.54 |
| 13_fear_threat | 0.49 |
| 23_asd_autism | 0.47 |
| 14_disease_ad_pd | 0.42 |
| 47_attention_attentional_target | 0.40 |
| 32_pain_somatosensory_stimulation | 0.35 |
| 43_magnetic_mechanisms_human | 0.34 |
| 15_task_performance_cognitive | 0.33 |
| 12_women_men_sex | 0.33 |
| 29_stress_ptsd_trauma | 0.29 |

The top 20 terms from the NiMARE meta-analytic decoding of significant FC clusters using the NeuroSynth meta-analytic database.

**Supplemental Table 5.** Individual CAPS-5 symptom group contrasts of PACAP and resting-state rCMA FC, stratified by sex assigned at birth.

|  | **IH vs. High** | | **IH vs. Low** | | **High vs. Low** | |
| --- | --- | --- | --- | --- | --- | --- |
|  | *Male* | *Female* | *Male* | *Female* | *Male* | *Female* |
| PACAP | *t* = 0.79,  *p* = 0.710 | *t* = 0.57,  *p* = 0.834 | *t* = 0.26,  *p* = 0.963 | *t* = 1.24,  *p* = 0.432 | *t* = -0.90,  *p* = 0.639 | *t* = 0.80,  *p* = 0.706 |
| rCMA-POS | t = 2.22,  *p* = 0.028 | t = 2.36,  *p* = 0.020 | t = 2.31,  *p* = 0.023 | t = 2.91,  *p* = 0.004 | *t* = 0.12,  *p* = 0.902 | t = 0.79,  *p* = 0.430 |
| rCMA-Precun | *t* = 3.37,  *p* = 0.001 | *t* = 4.11,  *p* < 0.001 | *t* = 3.16,  *p* = 0.002 | *t* = 3.97,  *p* < 0.001 | *t* = -0.30,  *p* = 0.768 | *t* = 0.15,  *p* = 0.881 |
| rCMA-mPFC | *t* = 2.15,  *p* = 0.034 | *t* = 2.85,  *p* = 0.005 | *t* = 2.64,  *p* = 0.009 | *t* = 4.33,  *p* < 0.001 | *t* = 0.70,  *p* = 0.485 | *t* = 1.85,  *p* = 0.066 |

rCMA = right centromedial amgydala, POS = parietooccipital sulcus, mPFC = medial prefrontal cortex.

**Figure S1.** **Amygdala subregions of interest.** Anatomical masks of the centromedial (red) and basolateral (blue) amygdala subregions used for functional connectivity analyses. Masks were generated from the JuBrain Atlas.

**REFERENCES**

1.	Esteban O, Markiewicz CJ, Blair RW, Moodie CA, Isik AI, Erramuzpe A, et al. fMRIPrep: a robust preprocessing pipeline for functional MRI. Nat Methods. 2019;16:111–116.

2.	Pruim RHR, Mennes M, van Rooij D, Llera A, Buitelaar JK, Beckmann CF. ICA-AROMA: A robust ICA-based strategy for removing motion artifacts from fMRI data. Neuroimage. 2015;112:267–277.

3.	Whitfield-Gabrieli S, Nieto-Castanon A. Conn: a functional connectivity toolbox for correlated and anticorrelated brain networks. Brain Connect. 2012;2:125–141.

4.	Adams SW, Neylan TC, May V, Hammack SE, Ressler K, Inslicht SS. PACAP associated with precise PTSD and fear extinction response in women. Psychoneuroendocrinology. 2025;173:107375.
